# Identification of diverse viruses in upper respiratory samples in dromedary camels from United Arab Emirates

**DOI:** 10.1371/journal.pone.0184718

**Published:** 2017-09-13

**Authors:** Yan Li, Abdelmalik Ibrahim Khalafalla, Clinton R. Paden, Mohammed F. Yusof, Yassir M. Eltahir, Zulaikha M. Al Hammadi, Ying Tao, Krista Queen, Farida Al Hosani, Susan I. Gerber, Aron J. Hall, Salama Al Muhairi, Suxiang Tong

**Affiliations:** 1 Division of Viral Diseases, National Center for Immunization and Respiratory Diseases, Centers for Disease Control and Prevention, Atlanta, Georgia, United States of America; 2 Animal Wealth Sector, Abu Dhabi Food Control Authority, Abu Dhabi, United Arab Emirates; 3 Oak Ridge Institute for Science and Education, Oak Ridge, Tennessee, United States of America; 4 Health Authority Abu Dhabi, Abu Dhabi, United Arab Emirates; Division of Clinical Research, UNITED STATES

## Abstract

Camels are known carriers for many viral pathogens, including Middle East respiratory syndrome coronavirus (MERS-CoV). It is likely that there are additional, as yet unidentified viruses in camels with the potential to cause disease in humans. In this study, we performed metagenomic sequencing analysis on nasopharyngeal swab samples from 108 MERS-CoV-positive dromedary camels from a live animal market in Abu Dhabi, United Arab Emirates. We obtained a total of 846.72 million high-quality reads from these nasopharyngeal swab samples, of which 2.88 million (0.34%) were related to viral sequences while 512.63 million (60.5%) and 50.87 million (6%) matched bacterial and eukaryotic sequences, respectively. Among the viral reads, sequences related to mammalian viruses from 13 genera in 10 viral families were identified, including *Coronaviridae*, *Nairoviridae*, *Paramyxoviridae*, *Parvoviridae*, *Polyomaviridae*, *Papillomaviridae*, *Astroviridae*, *Picornaviridae*, *Poxviridae*, and *Genomoviridae*. Some viral sequences belong to known camel or human viruses and others are from potentially novel camel viruses with only limited sequence similarity to virus sequences in GenBank. A total of five potentially novel virus species or strains were identified. Co-infection of at least two recently identified camel coronaviruses was detected in 92.6% of the camels in the study. This study provides a comprehensive survey of viruses in the virome of upper respiratory samples in camels that have extensive contact with the human population.

## Introduction

Camels have been associated with human civilization for thousands of years as a form of transportation of people and goods, a source of meat, milk and wool, and a player in recreational racing in the Middle East. Dromedary camels (camels), also known as Arabian or one-humped camels, inhabit the Middle East and parts of Africa and constitute 94% of all the camels on earth [1]. Like other animals, camels are frequently infected with viruses, but the risk of camel-to-human spread was thought to be low until the recent emergence of Middle East respiratory syndrome coronavirus (MERS-CoV), apparently from camels [2–4]. Before 2013, viruses of at least ten virus families, including *Nairoviridae*, *Paramyxoviridae*, *Flaviviridae*, *Herpesviridae*, *Papillomaviridae*, *Picornaviridae*, *Poxviridae*, *Orthomyxoviridae*, *Reoviridae* and *Rhabdoviridae*, have been found to infect dromedary and Bactrian camels [5–13].

MERS-CoV was first identified in the Kingdom of Saudi Arabia (KSA) in 2012 in a patient with acute respiratory distress syndrome [14], and later, camels were recognized as an animal source of MERS-CoV infection in humans and likely to be a major reservoir host for MERS-CoV. The discovery of widespread MERS-CoV infection in camels on the Arabian Peninsula has intensified the search for viruses in camels. In 2014, a human OC43-related betacoronavirus 1 species named dromedary camel coronavirus HKU23 was identified in camels [15]. In 2016, another coronavirus, camel alphacoronavirus (alpha-CoV), was identified in camels, sharing around 90% nucleotide (nt) identity with the human alpha-CoV 229E [16, 17]. Until now, three coronaviruses have been identified in camels: MERS-CoV, human OC43-related camel coronavirus HKU23 and human 229E-related camel alpha-CoV. Camels have been suggested to play an important role in the emergence of MERS-CoV and the evolution of coronaviruses (CoVs) [16, 17].

Methods for virus identification in camels have often been based on traditional serological methods and virus isolation, and more recently, molecular detection methods, such as RT-PCR and DNA sequencing have been applied. For example, novel camel enteroviruses and camel astroviruses were identified using RT-PCR [18, 19]. The advancement of next generation sequencing (NGS) technologies and metagenomic analysis have facilitated large-scale detection of known and novel viruses from a wide variety of animals such as bats [20, 21], cats [22], ducks [23], pigs [24] and other mammals [25]. NGS was also used to facilitate full genome sequencing of CoVs in nasal swab samples from camels in Saudi Arabia and Kenya [16, 17]. The first and only metagenomic study of the camel virome was carried out by Woo *et al*. on a pool of fecal samples from 203 adult camels [26]. Woo *et al*. obtained a total of about 29.25 million 151-bp sequence reads that were assembled into 159,388 contigs. 4.6% of these contigs were viral sequences. More detailed analysis of this pooled fecal sample revealed the presence of known as well as novel species of mammalian viruses from the viral families *Picobirnaviridae*, *Circoviridae*, *Picornaviridae*, *Parvoviridae*, *Astroviridae*, *Hepeviridae*, *Reoviridae and Caliciviridae*.

To date, the United Arab Emirates (UAE) has reported the third highest number of human MERS cases to the World Health Organization [27]. MERS-CoV RNA and antibodies have been detected in camels from UAE [28–30]. Molecular epidemiology investigations of the MERS cases and outbreaks in 2013–2014 in Abu Dhabi, UAE identified direct links between viruses recovered from human cases and camel MERS-CoV sequences sampled from UAE (publication submitted). In 2015, WHO reported a German MERS patient who was believed to have contracted the virus from an infected camel during a visit to a live animal market in the UAE in February, 2015 [31]. MERS-CoV testing and sequencing in camels from the same market identified almost identical MERS-CoV sequences and also showed a much higher MERS-CoV positive rate (publication submitted). Taken together, these results suggested that camels at UAE live animal markets may actively participate in the spread of MERS-CoV among animals and/or humans and that camel exposure is a risk factor for zoonotic transmission of MERS-CoV and potentially other viral agents.

In this study, nucleic acid extracts of nasopharyngeal (NP) swab samples from 108 MERS-CoV-positive camels in Abu Dhabi were subjected to metagenomic sequencing analysis. The goal of this study is to apply unbiased shotgun sequencing to hunt for viruses in camels, particularly those with potential for zoonotic transmission.

## Materials and methods

### Camel samples and nucleic acid extraction

NP swab samples were collected from 376 apparently healthy camels as part of a MERS-CoV surveillance study carried out at a live animal market in Abu Dhabi, UAE. Collection of samples from camels performed in this study was approved by the CDC Institutional Animal Care and Use Committee (protocol number 2702HALMULX). The age, sex, origin, pen number and the initial MERS-CoV qPCR result of the 108 MERS-CoV-positive camels that were further analyzed at CDC are shown in S1 Table. Total ribonucleic acid (RNA) was extracted and purified using the EZ1 Virus Mini Kit 2.0 (QIAGEN) and stored at -80°C.

### Pre-amplification, library construction and Illumina HiSeq sequencing

To minimize the issue of contamination, we have run sample processing, pre-amplification, library preparation, and sequencing in a contained hood in separate rooms. Each RNA sample was reverse-transcribed and amplified with a modified random amplification (Round AB) protocol as described previously [32]. PCR amplicons obtained from the pre-amplification were purified with Agencourt AmPure XP (Beckman Coulter) and subsequently fragmented by sonication using a Covaris LE220 focused-ultrasonicator (Covaris) in order to achieve efficient library generation. Library construction for Illumina sequencing was carried out for each sample individually using NEB Ultra II DNA Library Prep Kit (New England Biolabs) with dual index barcoding according to the manufacturer’s instructions. Library construction was done either manually or on an automated NGS workstation, Zephyr G3 NGS Workstation (PerkinElmer). Quality of the libraries generated was evaluated using a 2200 TapeStation Nucleic Acid System (Agilent Technologies) and library DNA concentrations were measured using Qubit 2.0 fluorometer (Thermo Fisher Scientific). The libraries were then normalized, pooled, and sequenced on an Illumina HiSeq 2500 (2x125) or MiSeq (2x300) instrument, aiming for 5–10 million pair-end reads per sample following the company’s standard protocols.

### NGS data analysis

Data was primarily analyzed using the SURPI pipeline with standard parameters [33]. Briefly, Illumina reads were trimmed and classified by read mapping to the NCBI nt database. Unclassified reads were de novo assembled, and the resulting contigs and unassembled reads were classified based on translated nucleotide similarity to the NCBI NR database. Reads were binned by taxonomy, and the viral reads and contigs classified by either method were assigned, per genus per sample, to the most similar available reference in that genus, using BLAST. In cases where there were more than one virus identified per genus, reads from that genus were mapped using BWA-MEM to an index of the candidate references. For positive hits, we used relative read count thresholds based on an empirical overall barcode contamination rate (0.01%) to exclude hits with low viral read counts when other samples with high read counts for that taxon were sequenced on the same HiSeq lane. In addition, positive hits from the SURPI summary report with fewer than 100 reads were aligned to the NCBI nt database using BLASTN and manually reviewed to ensure the accuracy of virus classification before the final calls were made. The positive samples that had low number of viral reads from NGS were further confirmed by PCRs followed by amplicon sequencing (S2 Table). In addition, all samples were screened for coronaviruses [34], parvoviruses, orthonairoviruses, paramyxoviruses [35], polyomaviruses [36], and astroviruses by PCR for better sensitivity. When possible, BWA-MEM [37] and samtools [38] were used to generate consensus sequences from reads and contigs for phylogenetic analysis and further genomic analysis.

### Phylogenetic analysis

The longest continuous contig of each unique viral sequence recovered from this study was selected as a representative for each phylogenetic tree except for polyomavirus where the longest coding sequence for large T antigen was used. Multiple sequence alignment was done by MAFFT Version 7 (http://mafft.cbrc.jp/alignment/server). A substitution model test was done for each alignment, and the best model, general time reversible nucleotide substitution model with gamma correction (GTR+G), was used to construct maximum likelihood (ML) phylogenetic trees with 100 bootstrap replications using MEGA Version 6.06. Taxonomic cladograms (S1 Fig) were generated using the phylo_dot_plot.pl script [39]. The dataset used for each phylogenetic reconstruction is provided in S1–S6 Files.

### Data availability

The data generated in this study is available online. Illumina read data has been deposited in the NCBI Sequence Read Archive as Project PRJNA396214. Complete or partial genomes are available in GenBank as follows: camel alpha-CoV Abu Dhabi B38: MF593473; camel coronavirus HKU23 Abu Dhabi B101: MF593476; camel PIV3 Abu Dhabi B49: MF593477; camel PIV4 Abu Dhabi B89: MF593478; camel bocavirus 3 Abu Dhabi B41: MF593479; camel CCHFV Abu Dhabi B77: MF593474; camel polyomavirus Abu Dhabi B83: MF593475.

## Results

### Metagenomic analysis of camel NP swab samples

A total of 108 individual barcoded libraries from camel NP swab RNA samples were subjected to shotgun Illumina sequencing. In total, 846.72 million high quality sequence reads were obtained from 108 samples, averaging 7.8 million reads per sample. Among the total reads, 0.34% (2.88 million) were classified as viral sequences by SURPI. Bacterial and eukaryotic sequences accounted for 60.5% (512.63 million) and 6% (50.87 million), respectively. The remaining 33.1% (280.33 million) of the reads did not match any sequences in the NCBI database by nucleotide similarity search.

**Fig 1:**
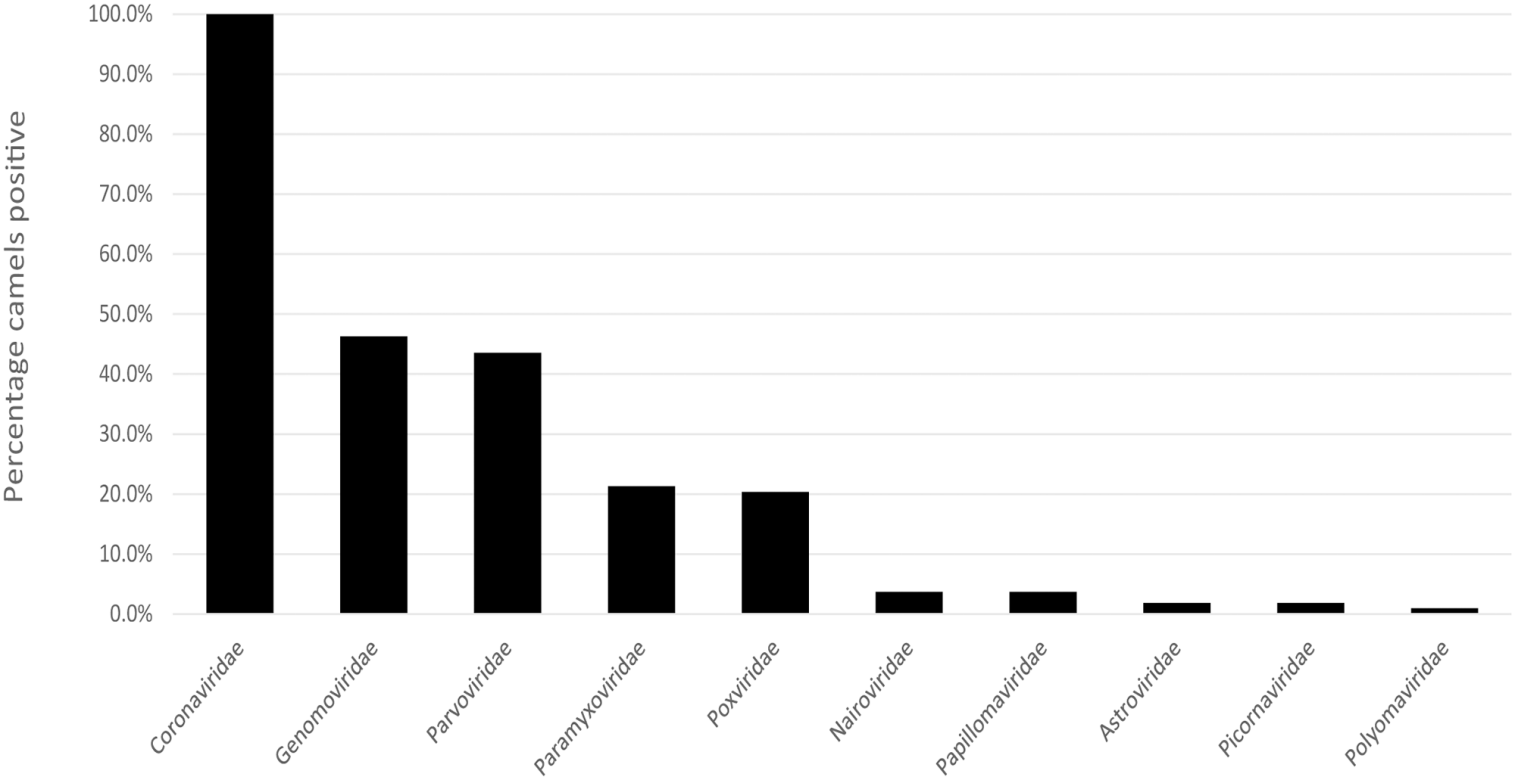
Prevalence of mammalian virus families identified in camels from a single live animal market in Abu Dhabi in the United Arab Emirates (UAE). The percentage of camels that were positive for each viral family is depicted by the bar graph.

SURPI initially identified viral signature sequences belonging to 33 viral families involving 85 genera. In this work, we focused on viral reads associated with mammals; bacteriophages and viruses pertaining primarily to plants, fungi, insects, and amoebas are not further discussed. Out of 2.88 million high-quality viral reads, 2.36 million (81.9%) were initially classified as mammalian viruses by nucleotide similarity and were further checked by reference-based mapping, BLASTN, and/or PCR to eliminate the potentially false positive viral reads. A cladogram that shows all of the virus genera found in camels is shown in S1 Fig. A total of 10 viral families covering 13 genera of mammalian viruses were confirmed (Table 1), and the detection rate of each viral family in the camel population is shown in Fig 1. As expected, *Coronaviridae* was detected in each of the 108 samples as these samples had been pre-screened as MERS-CoV positive. In addition to *Coronaviridae*, *Genomoviridae*, *Parvoviridae*, *Paramyxoviridae*, and *Poxviridae* were among the top five viral families identified at a high rate (Fig 1). The genera of the viruses detected in each sample by shotgun sequencing are listed in S1 Table. Of the 108 samples, 106 samples (98.1%) were positive for two or more mammalian virus genera and 78 (72.2%) were positive for three or more mammalian virus genera (S1 Table).

**Table 1:** Mammalian viruses detected in camels from a single live animal market in the United Arab Emirates.

Family	Genus	Virus (best match)	No. positive samples (%)	nt identity*	The longest contig (length)	Genome coverage length^&^
*Astroviridae*	*Mamastrovirus*	Dromedary astrovirus	2 (1.9%)	98%	orf1ab (370 nt)	370 nt
*Nairoviridae*	*Orthonairovirus*	Crimean-Congo hemorrhagic fever virus	4 (3.7%)	89%	RdRP/L (531 nt)	1363 nt
*Coronaviridae*	*Alphacoronavirus*	Camel alphacoronavirus	100 (92.6%)	99.9%	Genome (27389 nt)	27389 nt
	*Betacoronavirus*	MERS coronavirus	108 (100%)	99.7%	Genome (30056 nt)	30112 nt
		Camel coronavirus HKU23	25 (23.1%)	99.7%	Genome (30994 nt)	31007 nt
*Genomoviridae*	*Gemykrogvirus*	Caribou feces-associated gemycircularvirus	50 (46.3%)	100%	Genome (2232 nt)	2232 nt
*Papillomaviridae*	*Deltapapillomavirus*	Camelus dromedarius papillomavirus type 1	4 (3.7%)	98%	E2 (234 nt)	812 nt
*Paramyxoviridae*	*Respirovirus*	Bovine parainfluenza virus 3	22 (20.4%)	86%	RdRP/L (4575 nt)	14704 nt
	*Rubulavirus*	Human parainfluenza virus 4	1 (0.9%)	84%	RdRP/L (258 nt)	2239 nt
*Parvoviridae*	*Bocaparvovirus*	Canine bocavirus 3	40 (37.0%)	85%	NS1, NP1, VP1 (1905 nt)	4607 nt
	*Dependoparvovirus*	Adeno-associated virus	7 (6.5%)	97%	Capsid (647 nt)	2679 nt
*Picornaviridae*	*Kobuvirus*	Bovine kobuvirus	2 (1.9%)	92%	3D (195 nt)	205 nt
*Polyomaviridae*	*unclassified Polyomaviridae*	Sheep polyomavirus 1	1 (0.9%)	62%	Large T antigen, small T antigen (722 nt)	2600 nt
*Poxviridae*	*Orthopoxvirus*	Camelpox virus	10 (9.3%)	99%	Uracil-DNA glycosylase/UNG (163 nt)	923 nt
	*Parapoxvirus*	Orf virus	12 (11.1%)	96%	ORFV118 (244 nt)	2295 nt

* Based on the longest contig of each virus from all samples.

^&^ Based on the sample that has the longest genome coverage.

### Identification of previously known camel viruses

**Fig 2:**
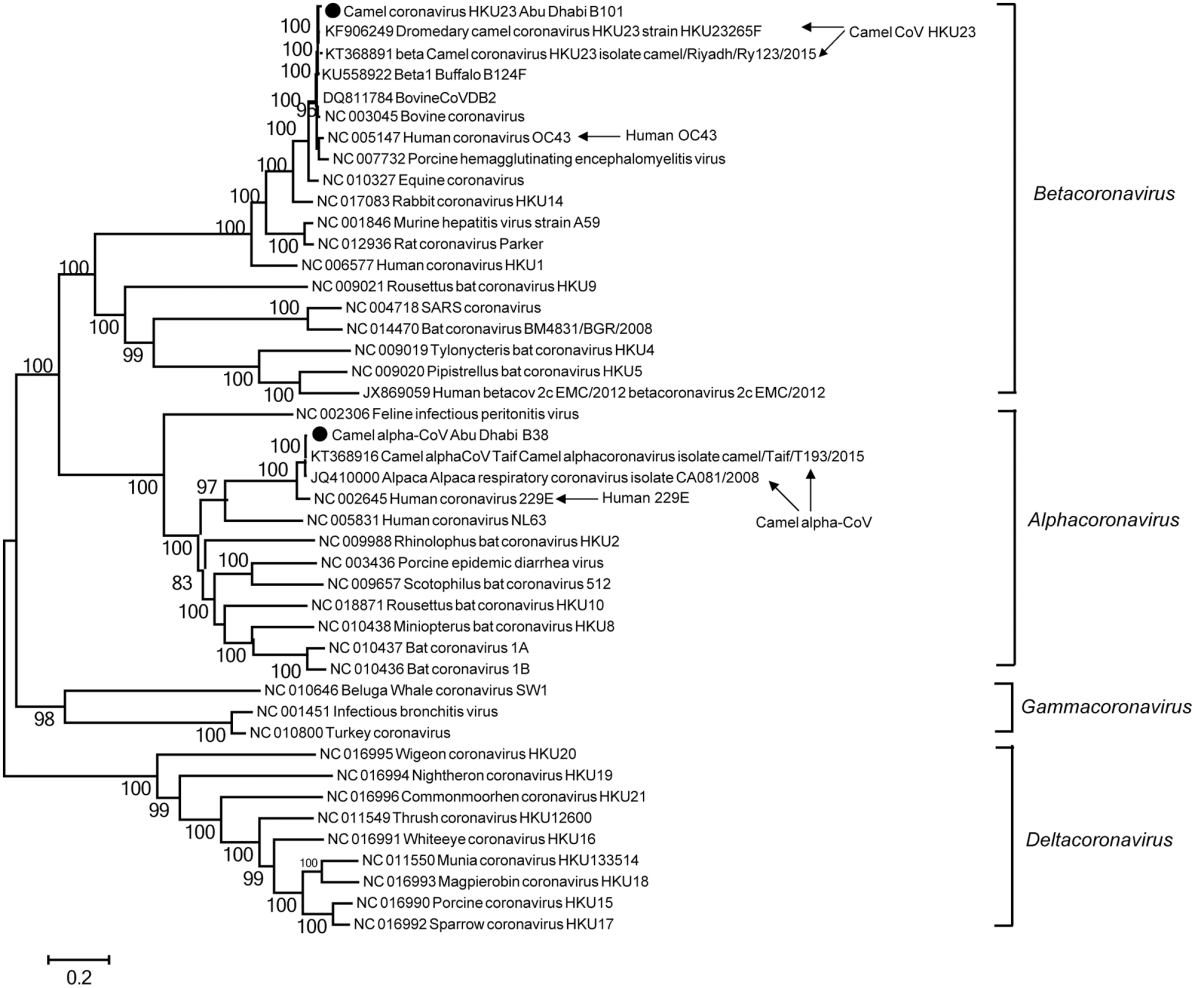
Phylogenetic analysis of the camel coronaviruses. Maximum likelihood phylogenetic tree was estimated using complete or near complete genomes from 42 previously published coronavirus sequences, one representative camel alpha-CoV Abu Dhabi full genome, and one representative camel CoV HKU23 Abu Dhabi genome (≥99.5% genome coverage) from this study. The four genera in *Coronavirinae* are labeled accordingly. Previously published camel alpha-CoV and camel CoV HKU23 as well as the human 229E and OC43 CoVs are indicated by arrows. Camel virus sequences identified in this study are highlighted by solid circles. The scale bar indicates the estimated number of nt substitutions per site and the bootstrap values (≥80) are indicated.

Because the camels in this study are from a MERS-CoV surveillance study, each of the 108 samples we analyzed harbored at least one coronavirus (Table 1, S1 Table). Full MERS-CoV genome sequences (≥99% complete) were obtained from shotgun sequencing in 27 samples which had relatively high viral loads (Ct from 17 to 26). The sequences of these 27 MERS-CoV genomes shared >99.7% identity with each other, and >99.5% identity compared to other published MERS-CoV sequences. In addition, we detected two other recently identified camel coronaviruses: camel coronavirus HKU23 in 25 camels (23.1%) and camel alpha-CoV in 100 camels (92.6%), named as camel coronavirus HKU23 Abu Dhabi and camel alpha-CoV Abu Dhabi, respectively. Out of 25 camel coronavirus HKU23 Abu Dhabi-positive samples, near full genome sequences (>85% of the length of the reference genome) were recovered from three samples with >99.7% nt identity among themselves. These sequences also shared ≥99.7% identity with the camel coronavirus HKU23 sequences identified previously in camels from Dubai, UAE [15] and Riyadh, Saudi Arabia [17]. Of the 100 camel alpha-CoV-positive samples, 23 near full genome sequences (≥95% complete) were recovered from 23 camels with >99.7% nt identity among themselves and ≥99.5% nt identity to the newly discovered camel alpha-CoV [17]. The relationship between the CoVs found in this study and the known camel CoVs is demonstrated by phylogenetic analysis (Fig 2). Notably, we observed a 92.6% co-detection rate of MERS-CoV and camel alpha-CoV (100 out of 108), and 22.2% of camels harbored all three CoVs (24 out of 108).

In two camels, we identified partial sequences of camel astrovirus (370 nt in orf1ab gene by PCR in one camel and 132 nt in orf1ab by NGS in the other camel) that shared 98% nt identity with the recently discovered dromedary astrovirus DcAstV-274 [19] (Table 1, S1 Table). Another four camels contained sequences similar to a papillomavirus (234 bp in E2 gene). Those sequences matched with 98% nt identity to *Camelus dromedarius* papillomavirus type 1 (Table 1, S1 Table).

Finally, we detected stretches of short sequences in 10 camels with high sequence homology to multiple regions of camelpox viruses (Table 1, S1 Table). The longest contig obtained was 163 bp long with 99% nt identity (Table 1). In 12 camels, there were stretches of short sequences with similarity to Orf virus, a parapoxvirus. The longest contig was 244 bp in length with 96% nt identity (Table 1, S1 Table).

### Identification of novel viral sequences

#### Parainfluenzaviruses

**Fig 3:**
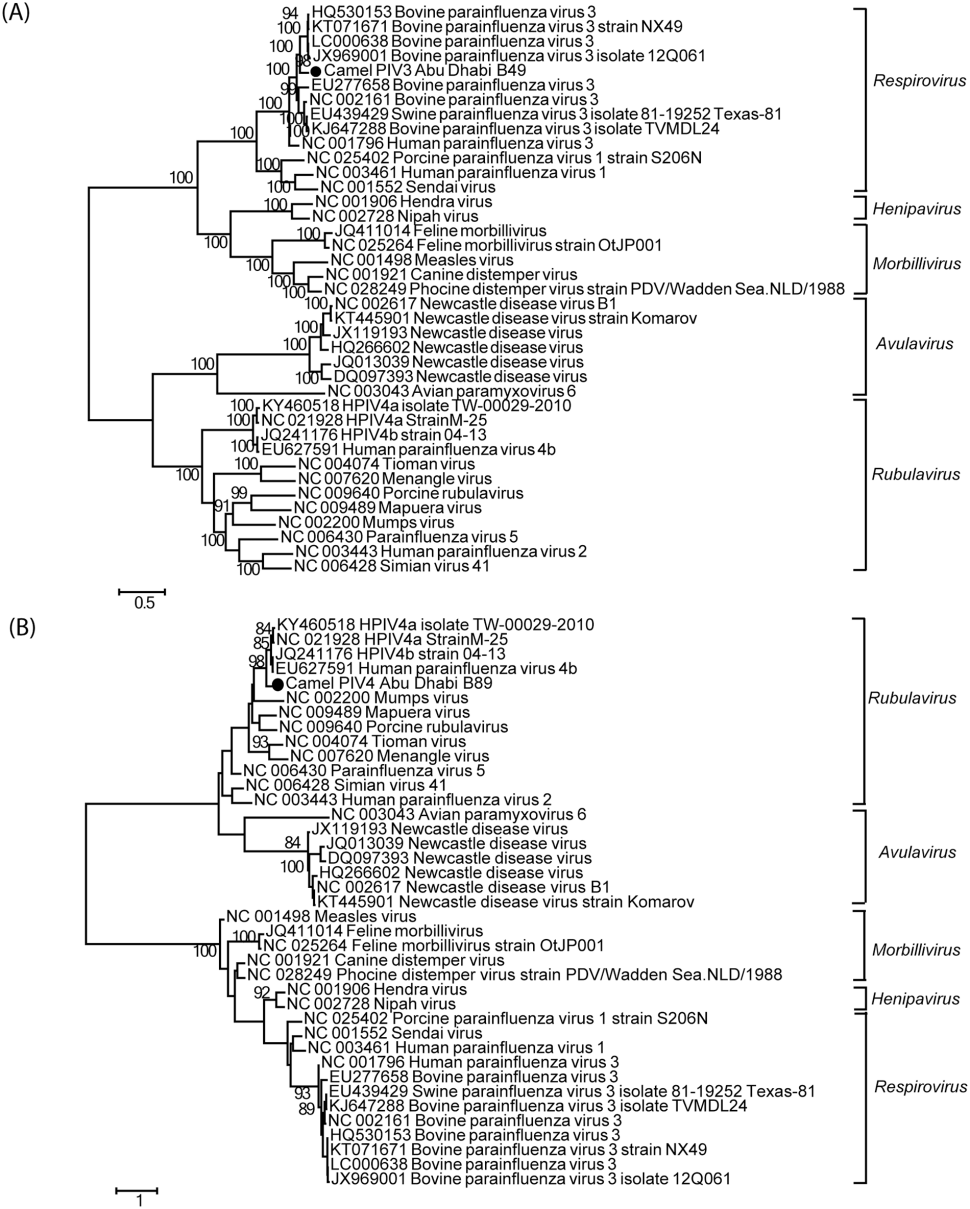
Phylogenetic analysis of the camel paramyxoviruses. Phylogenetic relationships are shown using maximum likelihood phylogenetic trees based on 38 paramyxovirus reference sequences and (A) the representative camel PIV3 Abu Dhabi sequence (4575 nt in L gene), or (B) the camel PIV4 Abu Dhabi sequence (258 nt in L gene) from this study. Five genera in *Paramyxoviridae* are labeled accordingly. Camel virus sequences identified in this study are highlighted by solid circles. The scale bar indicates the estimated number of nt substitutions per site and the bootstrap values (≥80) are indicated.

Twenty-two camels out of 108 (20.4%) yielded viral reads that matched to bovine parainfluenza virus 3 (BPIV3), in the genus *Respirovirus* (Table 1, S1 Table). We recovered 86% and 95% of the full genome from two of these samples. The camel PIV3 viral sequences from this study are >99% identical to each other and showed an overall 86% nt identity to BPIV3, suggesting a new strain of BPIV3 in camels which was tentatively named camel parainfluenza virus 3 (camel PIV3) Abu Dhabi. Genome analysis of the near full genome sequence (95% genome length) revealed similar ORFs and genome structure compared to BPIV3. Phylogenetic analysis using the longest contig sequence confirmed the close relationship of camel PIV3 with multiple BPIV3 strains (Fig 3A).

We also identified stretches of short sequences, 2239 nt in total, in one camel with homology (84% nt identity by the longest contig) to human parainfluenza virus 4 (HPIV4) in the genus *Rubulavirus* (Table 1, S1 Table). These stretches of short sequences were from the genes coding for nucleocapsid, matrix, fusion, and RdRP proteins. Based on the homology of the limited available sequences, it could represent a potentially new strain or a new species depending on the full genome sequence, thus it was tentatively named camel parainfluenza virus 4 (camel PIV4) Abu Dhabi (Fig 3B).

#### Bocaparvoviruses

**Fig 4:**
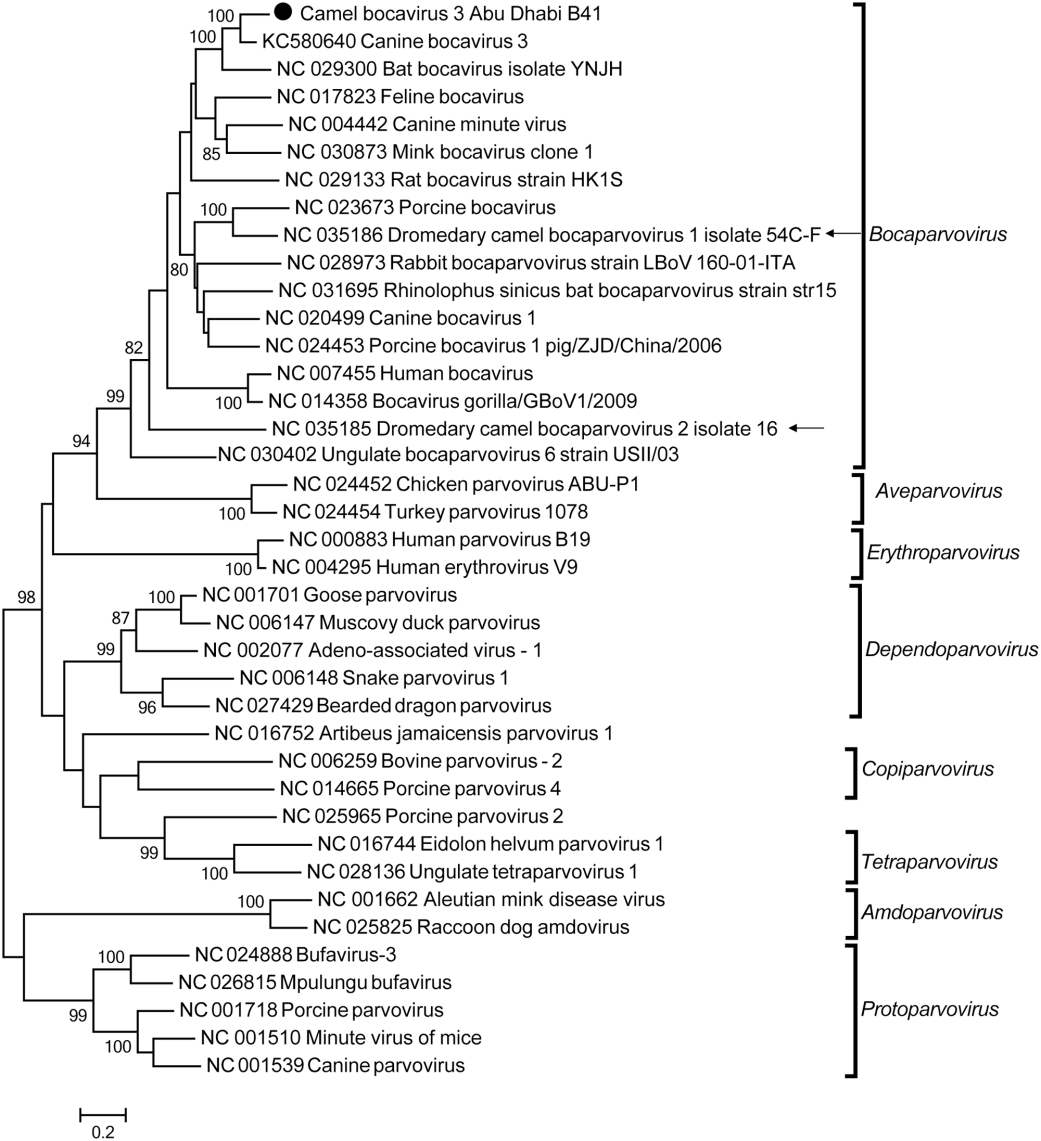
Phylogenetic analysis of camel bocavirus 3 Abu Dhabi. Maximum likelihood phylogenetic tree was estimated based on a 1905 nt-long contig sequence from one representative camel bocavirus 3 Abu Dhabi sequence detected in this study and 38 previously published sequences in *Parvovirinae*. The eight genera in *Parvovirinae* are labeled accordingly. The two known camel bocaparvoviruses are indicated by arrows. The camel bocaparvovirus identified in this study is highlighted by a solid circle. The scale bar indicates the estimated number of nt substitutions per site and the bootstrap values (≥80) are indicated.

In 40 camels (37%), we identified viral sequences that belong to the *Bocaparvovirus* genus in the family *Parvoviridae* (Table 1, S1 Table). Among these samples, three had sequences that covered >70% of the genome. These sequences were most similar to canine bocavirus 3, an unclassified bocaparvovirus which was discovered from a dog liver [40]. The three partial genome sequences are >99% similar to each other and share only 85% nt identity with canine bocavirus 3, suggesting a potentially new virus strain in camels. Interestingly, the camel bocavirus 3 from this study had only 50–60% nt identity to the recently identified camel bocaparvoviruses from dromedary faecal samples in Dubai [41]. We tentatively named this virus camel bocavirus 3 Abu Dhabi. The recovered genome sequences included sequences from three bocavirus ORFs: NS1, NP1, and VP1. Phylogenetic analysis based on the longest contig (1905 nt in NS1, NP1 and VP1 genes) demonstrated that the camel bocaviruses cluster with canine bocavirus 3, together with porcine, feline, mink, and other canine bocaviruses (Fig 4).

#### Orthonairoviruses

**Fig 5:**
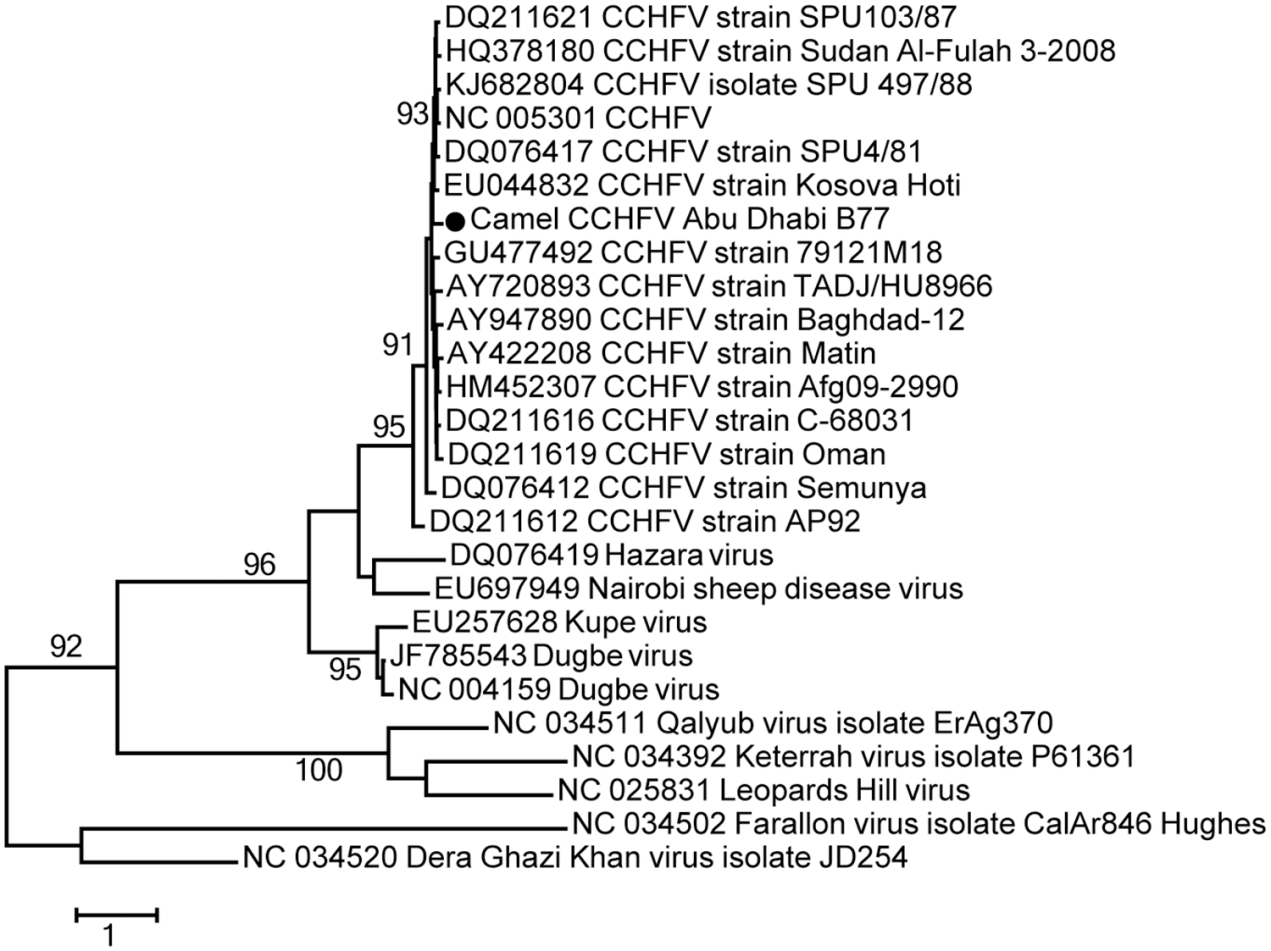
Phylogenetic analysis of camel CCHFV Abu Dhabi. Maximum likelihood phylogenetic tree was estimated from the longest contig (531 nt in L segment) obtained and 25 previously published L segment sequences in the *Orthonairovirus* genus. The representative camel CCHFV virus sequence identified in this study is highlighted by a solid circle. The scale bar indicates the estimated number of nt substitutions per site and the bootstrap values (≥80) are indicated.

We identified sequence reads in four camels that shared homology with Crimean-Congo hemorrhagic fever virus (CCHFV), a member of *Orthonairovirus* genus in the family *Nairoviridae* (Table 1, S1 Table). About 10% of the L segment homolog, 1077–1363 nt in total length, was recovered in each of three samples. These three samples were further confirmed by RT-PCR while the fourth one was identified only by RT-PCR. Among the three positive samples by NGS, a short (66nt-long) sequence fragment shared by two samples is identical between the two samples. Additionally, all four sequences share the same best match by BLASTN to CCHFV strain SPU103/87 while the longest contig shared 89% nt identity. These positives were all confirmed by RT-PCR. The lower sequence identity of available genome sequences suggests a potentially new CCHFV strain in camels, tentatively named camel CCHFV Abu Dhabi. Phylogenetic analysis based on the longest contiguous fragment (531 nt) showed that camel CCHFV Abu Dhabi clusters with CCHFV strains from human and ticks, well separated from other orthonairoviruses (Fig 5).

#### Polyomavirus

**Fig 6:**
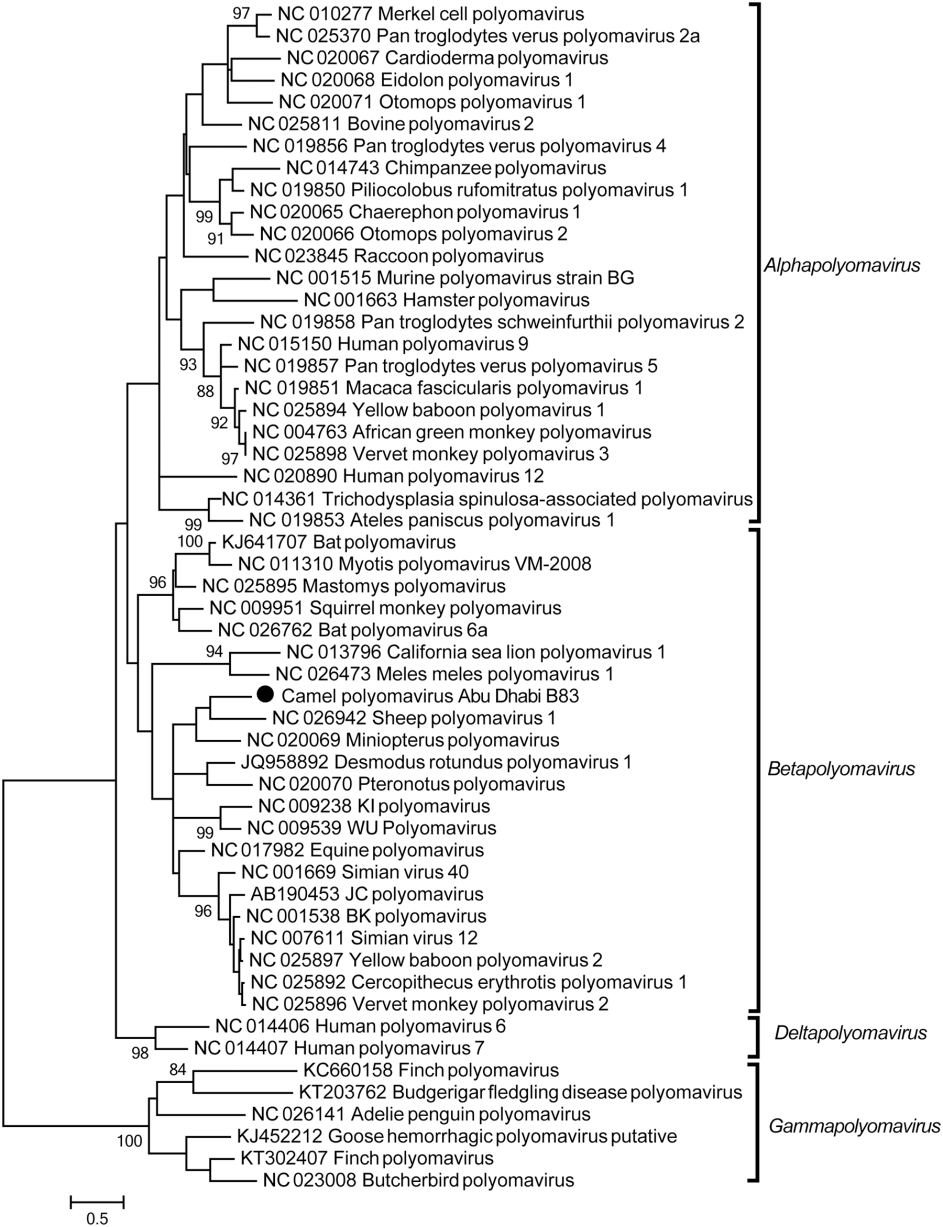
Phylogenetic analysis of camel polyomavirus Abu Dhabi. Maximum likelihood phylogenetic tree was estimated from the longest contiguous large T antigen coding sequence (384 nt) obtained and 53 previously published polyomavirus large T antigen sequences. The four genera in *Polyomaviridae* are labeled accordingly. The camel polyomavirus sequence identified in this study is highlighted by a solid circle. The scale bar indicates the estimated number of nt substitutions per site and the bootstrap values (≥80) are indicated.

Nucleotide sequences that were distantly related to known polyomaviruses were detected in one camel. Altogether, about half of the genome was covered by sequencing reads from this camel. The longest contig sequences covered partially the hypothetical genes large T antigen and small T antigen (722 bp in total) and capsid VP1 (411 bp), and shared about 62% and 69% nt identity, respectively, to a sheep polyomavirus, the nearest neighbor [42]. Since the suggested cutoff for new polyomavirus species is 85% genome-wide pairwise nucleotide identity [43], we suggest this virus is a potentially novel polyomavirus species, tentatively named camel polyomavirus Abu Dhabi. We further confirmed the positive result by PCR and subsequent Sanger sequencing. The phylogenetic analysis based on a 384 bp large T antigen coding fragment placed camel polyomavirus Abu Dhabi in a cluster with sheep polyomavirus 1 and several bat polyomaviruses (Fig 6).

### Detection of viral sequences previously found only in humans or non-camel animals

We identified sequences from several viruses that are known to infect humans or other animals, but previously had not been detected in camels. A total of 4904 reads aligned to viruses in the genus *Gemykrogvirus*, from 50 camels (46.3%) (Table 1, S1 Table). *Gemykrogviruses* are a newly recognized genus of the family *Genomoviridae* found initially in fungi [44, 45]. Two full genomes and one near-full genome (99%) were recovered from three camels, and the sequences were identical to caribou feces-associated gemycircularvirus [46]. In addition, sequences that were nearly identical to adeno-associated virus (AAV) were detected in seven camels (Table 1, S1 Table). We also detected reads related to kobuvirus, belonging to *Picornaviridae*, in two camels. The longest fragment obtained from both samples (195 nt) shares 92% nt identity to its closest neighbor, bovine kobuvirus strain SC1, an unclassified kobuvirus. This virus was tentatively called camel kobuvirus Abu Dhabi.

## Discussion

Because of the close association of camels with humans and the role played by camels in the emergence and transmission of MERS-CoV, it has become increasingly clear that camels are a critical intermediary in the transmission of viruses to humans. Thus, it is important to understand the repertoire of viruses that camels harbor and their zoonotic potential. Here we report the first study of camel respiratory viromes. Our results demonstrate that camels carry a wide variety of mammalian viruses. Among the different viruses detected in this study, some were similar or nearly identical to previously described camel viruses (96–99.7% nt identity), such as camel MERS-CoV, camel alpha-CoV, camel CoV HKU23, dromedary astrovirus, *Camelus dromedarius* papillomavirus, Orf virus, and camelpox virus. We also identified viral sequences that were similar or identical (92–100% nt identity) to viruses known to infect humans or animals other than camels, such as AAV, kobuvirus, and caribou feces-associated gemycircularvirus. Some viruses identified in this study, such as camel CCHFV Abu Dhabi, camel PIV3 Abu Dhabi, camel PIV4 Abu Dhabi, and camel bocavirus 3 Abu Dhabi, only shared a moderate similarity to the known viruses (84–89% nt identity), and are potentially novel. Complete genome sequencing of these viruses including camel CCHFV Abu Dhabi and camel PIV4 Abu Dhabi would further ascertain whether they should be considered novel strains or novel species. Finally, we identified sequences of a novel camel polyomavirus, camel polyomavirus Abu Dhabi most closely related to a sheep polyomavirus, with 62–69% nt identity in different gene regions.

Camel alpha-CoV has been very recently found to co-circulate with MERS-CoV in camels from Saudi Arabia with a coinfection rate of 56.6% by one study [17] and 3.5% by another [16]. In this study of camels from a single live animal market in UAE, we identified co-infection of camel alpha-CoV and MERS-CoV at a significantly higher rate of 92.6%, and co-infection of camel alpha-CoV, camel CoV HKU23 and MERS-CoV in 22.2% camels. This higher co-infection rate observed could be due to different camel population tested or sampling bias. Previous studies have shown that inter- and intra-coronavirus species recombination events happen frequently and are important for coronavirus evolution [47, 48]. Natural recombination between distantly related African bat coronaviruses resulted in a NL63-like virus, an ancestor of the human pathogen, human CoV NL63, suggesting that inter-species recombination may play an important role in CoV evolution and the emergence of novel CoVs with zoonotic potential [49]. In camels, recombination events between different MERS-CoV lineages in camels have been reported [17, 50]. A more recent study provided evidence for a cross-viral family recombination of a bat coronavirus and a reovirus [51]. The observation that camels are co-infected with different coronaviruses at a high rate further supports that camels may be a rich source for recombination and emergence of novel coronaviruses.

Several camel viruses we found were closely related to viruses in other animal species, particularly cattle, sheep, and dogs. The close relationships of these camel viruses to viruses of other species suggest that these viruses may have the capacity to infect multiple species or that they have only recently diverged after cross-species transmission, highlighting the potentially important role that camels play in emergence and evolution of these viruses. This phenomenon might also be a reflection of the fact that these camels were sampled from a live animal market, which houses several other species, such as cattle, sheep, and goats routinely, as well as other animals (chickens, dogs, cats) sporadically. As a result, this camel population may have been particularly prone to exposure to viruses from other species. If these viruses are actively replicating in camels, it is possible that they may act as intermediate hosts, or “staging grounds,” for these viruses, similar to what has been seen with civets for severe acute respiratory syndrome coronavirus (SARS-CoV) [52]. Interestingly, we identified a paramyxovirus genome most closely related to the human virus HPIV4, for the first time in camels. It is possible that camels may be serving as an intermediate host for this and other viruses, and they may eventually facilitate zoonotic infection.

CCHFV is a tick-borne bunyavirus that causes Crimean-Congo hemorrhagic fever (CCHF) exclusively in humans [53]. A study on a CCHF outbreak in UAE detected CCHFV antibodies in about 6% of camels [54]. Although CCHFV antibodies have been repeatedly detected in asymptomatic livestock including camels, prior to this study there was no evidence of CCHFV genomic RNA detected directly from camels. CCHFV genomic RNA had only been detected from ticks collected from camel skin [54–56]. This study documents the first report of CCHFV genomic RNA in camels. Amplification by RT-PCR on separate aliquots of the NGS positive specimens (data not shown) provides orthogonal confirmation of the validity of the limited number of sequencing reads.

Parainfluenza virus 3 (PIV3) has been shown to cause widespread respiratory infections and outbreaks in mammals, including humans. In camels, however, PIV3 seems to play a minor role in causing clinically obvious respiratory diseases [57]. PIV3 has been studied as early as the 1960s primarily using serological methods [58, 59]. The seroprevalence of PIV3 in camels varies by country and study [57, 60–62], but PIV3 genome sequences in camels were previously not reported. In UAE, antibodies against PIV3 have been detected in 5.6% of apparently healthy racing camels [61]. In the current study, we detected BPIV3-like genetic material in multiple samples with high genome coverage, suggestive of high viral loads, in apparently healthy camels. This is consistent with previous studies showing camels have asymptomatic PIV3 infections [57].

Polyomaviruses are a family of DNA tumor viruses known to infect a wide spectrum of mammals and birds, including humans. With molecular and other technological improvements, the family *Polyomaviridae* has expanded dramatically from only a handful of polyomavirus species in the early 1980s to more than 100 genetically and biologically distinct polyomaviruses at present [63]. Nearly all of the more than 1400 complete genomes currently available in GenBank were deposited after 2000. Our current study has added a novel polyomavirus species to the family as well as added camels to the list of known polyomavirus hosts.

*Genomoviridae* is a viral family [64] consisting of several closely-related genera of small, circular, singe-stranded DNA genomes. These viruses have been sequenced from various environmental [65], plant- [66] and animal-associated samples [67]. Moreover, genomovirus DNA has been found in disease-associated human samples such as cerebrospinal fluid from patients with encephalitis [68, 69], pericardial fluid from a patient with pericarditis [70], HIV-positive blood samples [71], and plasma pools [72]. However, to date there is no direct or causative link identified. We observed a high positive rate and detected a significant number of sequences of genomoviruses from the genus *Gemykrogvirus* in camel respiratory samples. However, it is not clear whether these viruses actually replicate in camels.

Compared to the prior camel virome study of feces [26], the top five most frequently detected viral species present in the respiratory samples in this study (other than MERS-CoV) were camel alpha-CoV, caribou feces-associated gemycircularvirus, camel bocavirus 3, camel CoV HKU23, and camel PIV3. Coronaviruses and paramyxoviruses were not detected in the camel fecal virome study. On the other hand, picobirnaviruses, rotaviruses, noroviruses, hepatitis E virus, and several picornaviruses were found in the fecal virome study, but were not detected in the present study using the respiratory samples. These differences were likely due to different expected tissue tropisms of the viruses. Moreover, in this study we analyzed individual samples, rather than pools of samples, to capture frequencies of detection and sequence variation among the population for any given virus or viral group (Fig 1, Table 1, S1 Table). One potentially unusual characteristic about the camels in this study is that they were all positive for MERS-CoV with moderate to high viral loads. As a result, the virome in these camels may or may not be representative of the diversity of viruses in normal camels. As a next step, it will be important to look at samples from MERS-CoV-negative camels from the same market to understand the viral diversity and compare the relative prevalence of these viruses.

It is hypothesized that camels are the intermediate animal host that allowed the ancestral MERS-CoV in bats to cross the species barrier to enter humans. A similar mechanism was proposed for SARS-CoV: the bat ancestor SARS-CoV replicated in palm civets as an intermediate and gained the capacity to infect humans [52]. Anthony et al. reported the identification of a MERS-CoV-like CoV from a bat sampled in Uganda and suggested that this virus is unlikely to pose a zoonotic threat as is; rather, recombination was likely the critical step that allowed MERS-CoV to emerge in humans [73]. Additionally, a recent study suggested possible zoonotic transmission of camelpox virus from camels to humans [74]. Therefore, it is important to consider all the viruses that are present in camels and the risk they may pose to humans in close proximity. The data presented here describes camel viruses that are similar to those that are known or thought to cause disease in humans. Multiple viruses were detected in many of the camels, indicating widespread co-infection. The fact that these types of viruses can and do co-exist in the same animal further suggests that camels serve as hosts for the diversification and evolution of mammalian viruses and are intermediate in the transmission of viruses between animals and humans.

## Supporting information

S1 Fig Taxonomic distribution of found virus signatures by genus. Cladograms show the virus genera represented by at least 1 read per sample in the dataset. Circles located next to each genus are logarithmically proportional to the total number of reads from SURPI pipeline. The number of reads or samples is shown in parentheses. Cladograms were generated using the phylo_dot_plot.pl script.
(TIF)

S1 Table Camel samples and genera of mammalian viruses detected in this study. (PDF)

S2 Table PCR primers used for viruses detected in this study. (PDF)

S1 File Dataset used for phylogenetic analysis of camel coronaviruses Abu Dhabi for Fig 2. (FASTA)

S2 File Dataset used for phylogenetic analysis of camel PIV3 Abu Dhabi for Fig 3A. (FASTA)

S3 File Dataset used for phylogenetic analysis of camel PIV4 Abu Dhabi for Fig 3B. (FASTA)

S4 File Dataset used for phylogenetic analysis of camel bocavirus 3 Abu Dhabi for Fig 4. (FASTA)

S5 File Dataset used for phylogenetic analysis of camel CCHFV Abu Dhabi for Fig 5. (FASTA)

S6 File Dataset used for phylogenetic analysis of camel polyomavirus Abu Dhabi for Fig 6. (FASTA)
