# Supplementary figures and images for: Identification of diverse viruses in upper respiratory samples in dromedary camels from United Arab Emirates
Source: PLoS One. 2017 Sep 13;12(9):e0184718. doi: 10.1371/journal.pone.0184718 (PMC5597213; doi:10.1371/journal.pone.0184718)

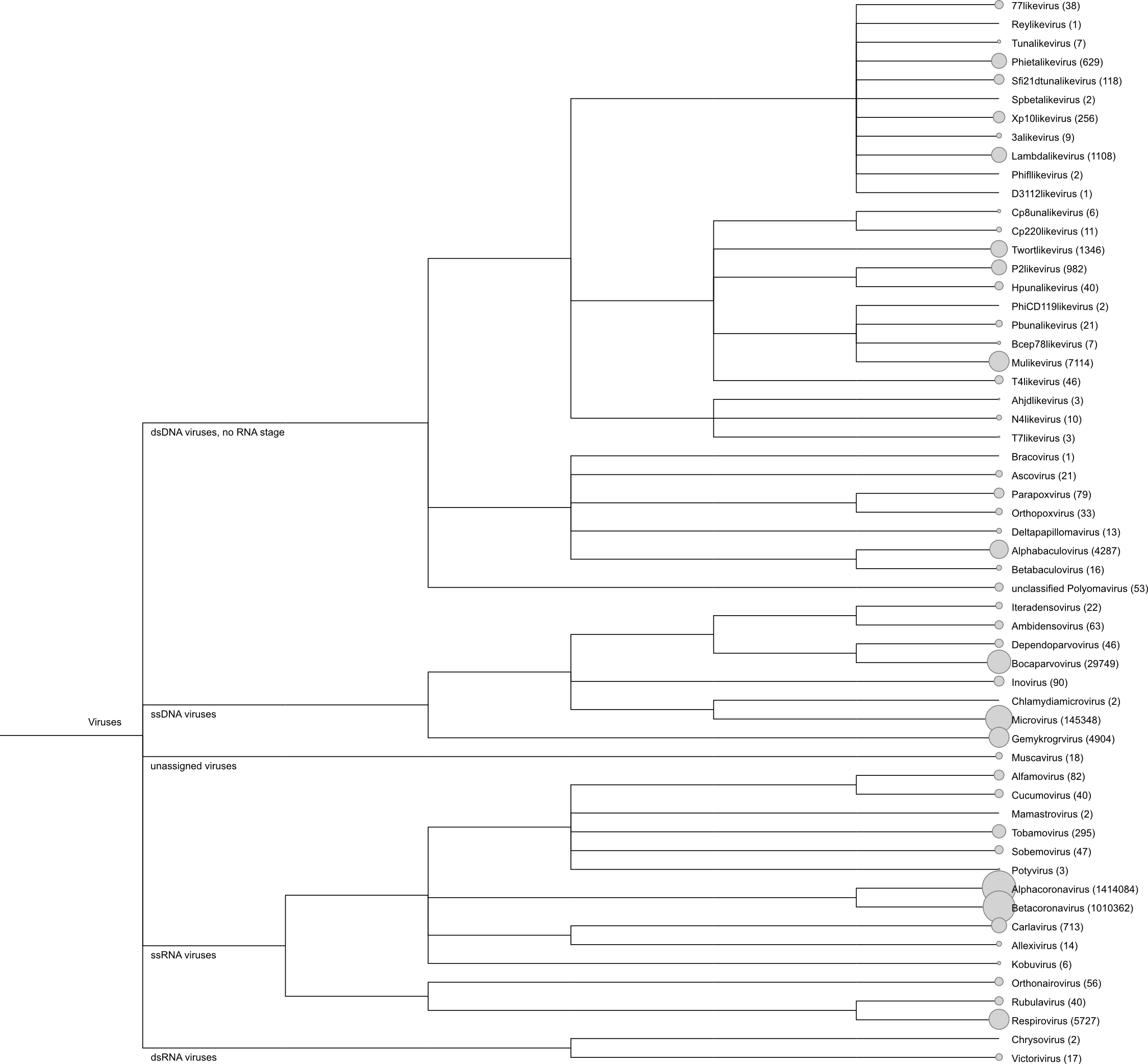

Supplement: S1 Fig — Cladograms show the virus genera represented by at least 1 read per sample in the dataset. Circles located next to each genus are logarithmically proportional to the total number of reads from SURPI pipeline. The number of reads or samples is shown in parentheses. Cladograms were generated using the phylo_dot_plot.pl script. (TIF) [file pone.0184718.s001.tif]
