# Supplementary material for: Identification of diverse viruses in upper respiratory samples in dromedary camels from United Arab Emirates
Source: PLoS One. 2017 Sep 13;12(9):e0184718. doi: 10.1371/journal.pone.0184718 (PMC5597213; doi:10.1371/journal.pone.0184718)
Supplement: S1 Table — (PDF) [file pone.0184718.s002.pdf]

**S1 Table. Camel sample and genera of mammalian viruses detected**

| Camels (from a live animal market in UAE) | | | | | | NGS results | | | | |
|---|---|---|---|---|---|---|---|---|---|---|
| **Sample ID** | **Age** | **Sex** | **Origin** | **Pen** | **MERS-CoV Ct** | **Total no of reads (million)** | Index seq 1 | Index seq 2 | NGS lane no. | **Viruses* detected (no. of reads, genome coverage)** |
| B1 | 1Year | Male | UAE | L42 | 31.22 | 10.19 | GAGATTCC | TAATCTTA | 1 | Alphacoronavirus (2, 0.65%), MERS-CoV (16, 4.99%) |
| B2 | 1Year | Male | UAE | L42 | 32.12 | 1.54 | GTGGCC | | M160108 | Alphacoronavirus (24, 13.16%), MERS-CoV (512, 75.90%) |
| B4 | 2-3 Years | Male | UAE | L49 | 18.98 | 9.44 | GAGATTCC | AGGCGAAG | 1 | Alphacoronavirus (9, 2.84%), MERS-CoV (30768, 99.98%), Orthopoxvirus (4, 0.19%) |
| B5 | 2-3 Years | Male | UAE | L49 | 28.85 | 8.92 | GAGATTCC | GGCTCTGA | 1 | Alphacoronavirus (3, 1.30%), MERS-CoV (47, 13.21%) |
| B6 | 2-3 Years | Male | UAE | L50 | 31.26 | 11.19 | GAGATTCC | CCTATCCT | 1 | MERS-CoV (33, 8.70%), Gemykrogrvirus (13, 36.35%), Orthopoxvirus (2, 0.11%) |
| B7 | 2-3 Years | Male | UAE | L50 | 30.95 | 8.39 | GAGATTCC | ATAGAGGC | 1 | Alphacoronavirus (24, 6.34%), MERS-CoV (203, 36.60%), camel coronavirus HKU23 (57, 12.68%), Gemykrogrvirus (5, 8.78%), Orthopoxvirus (3, 0.17%) |
| B8 | 2-3 Years | Female | UAE | L50 | 32.07 | 1.90 | ATGTCA | | M160115 | MERS-CoV (853, 75.85%) |
| B9 | 1Year | Female | UAE | L54 | 32 | 8.43 | GAGATTCC | TATAGCCT | 1 | Alphacoronavirus (18, 5.59%), MERS-CoV (198, 47.98%), camel coronavirus HKU23 (3, 0.92%), Gemykrogrvirus (288, 50.21%) |
| B10 | 1Year | Female | UAE | L54 | 18.62 | 8.26 | CGCTCATT | GTACTGAC | 1 | MERS-CoV (23369, 99.85%), Gemykrogrvirus (38, 70.83%) |
| B11 | 1Year | Female | UAE | L55 | 29.31 | 1.44 | GTGAAA | | M160115 | MERS-CoV (450, 61.10%) |
| B12 | 3 Months | Female | OMAN | L56 | 24.99 | 1.78 | GTTTCG | | M160108 | Alphacoronavirus (182924, 99.99%), MERS-CoV (21416, 99.91%) |
| B13 | 3 Months | Female | OMAN | L56 | 29.77 | 2.38 | CGTACG | | M160108 | Alphacoronavirus (365, 74.82%), MERS-CoV (1397, 95.31%), camel coronavirus HKU23 (1705, 93.06%) |
| B14 | 6-9 Months | Female | UAE | L57 | 29.19 | 8.31 | CGCTCATT | CAGGACGT | 1 | Alphacoronavirus (54, 15.92%), MERS-CoV (89, 22.02%), Gemykrogrvirus (51, 66.71%) |
| B15 | 6-9 Months | Female | UAE | L57 | 32.2 | 13.23 | CGCTCATT | TAATCTTA | 1 | Alphacoronavirus (14, 5.04%), MERS-CoV (13, 4.52%), camel coronavirus HKU23 (5, 1.72%), Gemykrogrvirus (191, 99.10%), Orthopoxvirus (4, 0.20%), Respirovirus (5, 2.39%) |
| B16 | 6-9 Months | Female | UAE | L57 | 33.29 | 9.26 | CGCTCATT | AGGCGAAG | 1 | Alphacoronavirus (4, 1.13%), MERS-CoV (19, 5.65%), Gemykrogrvirus (519, 100.00%), Orthopoxvirus (2, 0.06%) |
| B17 | 6-9 Months | Male | UAE | L59 | 31.82 | 11.68 | CGCTCATT | GGCTCTGA | 1 | Alphacoronavirus (4, 1.72%), MERS-CoV (8, 2.51%), camel coronavirus HKU23 (1, 0.38%), Bocaparvovirus (3, 3.87%), Gemykrogrvirus (7, 3.85%) |
| B18 | 6-9 Months | Male | UAE | L59 | 31.93 | 10.85 | CGCTCATT | CCTATCCT | 1 | Alphacoronavirus (2, 0.71%), MERS-CoV (4, 1.22%), Gemykrogrvirus (5, 13.93%) |
| B19 | 6-9 Months | Male | UAE | L59 | 31.09 | 7.76 | CGCTCATT | ATAGAGGC | 1 | Alphacoronavirus (12, 4.33%), MERS-CoV (10, 3.01%), Bocaparvovirus (2, 3.30%), Gemykrogrvirus (38, 20.52%), Orthopoxvirus (1, 0.06%) |
| B20 | 4 Months | Male | OMAN | L94 | 26.82 | 5.56 | CGCTCATT | TATAGCCT | 1 | Alphacoronavirus (11, 3.78%), MERS-CoV (2657, 97.29%), Dependoparvovirus (2, 2.66%) |
| B21 | 4 Months | Male | OMAN | L94 | 32.74 | 11.62 | TCCGGAGA | GTACTGAC | 1 | Alphacoronavirus (1, 0.43%), MERS-CoV (16, 5.55%) |
| B22 | 4 Months | Male | OMAN | L94 | 29.47 | 8.42 | TCCGGAGA | CAGGACGT | 1 | Alphacoronavirus (21, 6.15%), MERS-CoV (99, 23.40%), camel coronavirus HKU23 (12, 3.22%), Bocaparvovirus (13, 20.54%) |
| B23 | 4 Months | Male | OMAN | L94 | 32.55 | 10.24 | TCCGGAGA | TAATCTTA | 1 | Alphacoronavirus (518, 75.07%), MERS-CoV (5, 1.70%) |
| B24 | 4 Months | Male | OMAN | L94 | 32.29 | 7.73 | TCCGGAGA | AGGCGAAG | 1 | Alphacoronavirus (4, 1.49%), MERS-CoV (44, 10.88%), Respirovirus (5, 3.26%) |
| B25 | 7 years | Male | UAE | L91 | 31.4 | 12.32 | TCCGGAGA | GGCTCTGA | 1 | Alphacoronavirus (3, 0.95%), MERS-CoV (9, 2.11%), Gemykrogrvirus (3, 9.05%), Respirovirus (1, 0.51%) |
| B26 | 2-6 Years | Male | UAE | L90 | 21.75 | 7.64 | TCCGGAGA | CCTATCCT | 1 | Alphacoronavirus (1, 0.45%), MERS-CoV (3309, 99.13%) |
| B27 | 2-6 Years | Male | UAE | L90 | 32.54 | 10.69 | TCCGGAGA | ATAGAGGC | 1 | Alphacoronavirus (85, 24.67%), MERS-CoV (11, 3.19%), Bocaparvovirus (5, 7.46%), Gemykrogrvirus (10, 33.69%) |
| B28 | 8 Years | Male | UAE | L86 | 32.23 | 10.59 | TCCGGAGA | TATAGCCT | 1 | Alphacoronavirus (5, 1.65%), MERS-CoV (83, 20.69%) |
| B29 | 1 Month | Male | UAE | L85 | 32.2 | 4.90 | ATTACTCG | GTACTGAC | 1 | Alphacoronavirus (11, 3.99%), MERS-CoV (9, 3.18%), Bocaparvovirus (23, 2.41%), Dependoparvovirus (44, 57.79%), Respirovirus (4, 2.09%) |
| B30 | 6 Years | Female | UAE | L82 | 32.5 | 9.68 | ATTACTCG | CAGGACGT | 1 | Alphacoronavirus (4352, 96.75%), MERS-CoV (198, 31.44%), Gemykrogrvirus (1402, 34.95%), Respirovirus (44, 13.80%) |
| B31 | 4 Months | Male | UAE | L80 | 30.56 | 7.89 | ATTACTCG | TAATCTTA | 1 | Alphacoronavirus (2966, 98.34%), MERS-CoV (164, 28.97%), Gemykrogrvirus (24, 40.17%), Respirovirus (22, 8.82%) |
| B32 | 4 Months | Male | UAE | L80 | 33.24 | 5.06 | ATTACTCG | AGGCGAAG | 1 | Alphacoronavirus (15, 4.38%), MERS-CoV (29, 7.94%), Respirovirus (3302, 86.41%) |
| B33 | 6 Months | Male | OMAN | L78 | 25.5 | 1.40 | GTGGCC | | M160115 | Alphacoronavirus (3240, 96.56%), MERS-CoV (660, 75.37%) |
| B34 | 6 Months | Female | OMAN | L78 | 31.81 | 5.52 | ATTACTCG | GGCTCTGA | 1 | Alphacoronavirus (75, 19.79%), MERS-CoV (13, 3.60%) |
| B35 | 6 Months | Female | OMAN | L78 | 32.9 | 5.51 | ATTACTCG | CCTATCCT | 1 | Alphacoronavirus (106, 29.33%), MERS-CoV (29, 8.80%), Respirovirus (3, 2.27%) |
| B36 | 4 Months | Male | UAE | L74 | 33.29 | 9.38 | ATTACTCG | ATAGAGGC | 1 | Alphacoronavirus (35, 10.08%), MERS-CoV (22, 5.73%), Gemykrogrvirus (9, 30.29%) |
| B37 | <1 year | Male | OMAN | 77 | 19.29 | 4.48 | ATTACTCG | TATAGCCT | 1 | Alphacoronavirus (63734, 99.99%), MERS-CoV (73639, 99.89%), Gemykrogrvirus (2, 5.56%) |
| B38 | <1 year | Male | OMAN | 77 | 22.51 | 13.20 | GAATTCGT | GGCTCTGA | 2 | Alphacoronavirus (69762, 100.00%), MERS-CoV (7372, 99.87%) |
| B39 | <1 year | Male | OMAN | 77 | 24.07 | 13.73 | GAATTCGT | CCTATCCT | 2 | Alphacoronavirus (91548, 99.96%), MERS-CoV (3601, 99.12%), Mamastrovirus (2, 2.09%) |
| B40 | <1 year | Male | OMAN | 77 | 24.98 | 4.58 | GAATTCGT | ATAGAGGC | M160304 | Alphacoronavirus (3326, 99.42%), MERS-CoV (119, 32.69%) |
| B41 | <1 year | Male | OMAN | 77 | 28.22 | 2.93 | GTTTCG | | M160115 | Alphacoronavirus (41, 13.67%), MERS-CoV (2316, 94.88%), camel coronavirus HKU23 (18, 6.50%), Bocaparvovirus (5885, 87.29%) |
| B42 | <1 year | Male | UAE | 56 | 27.81 | 2.11 | GAGTGG | | M160108 | Alphacoronavirus (40, 19.55%), MERS-CoV (986, 91.70%), camel coronavirus HKU23 (10, 2.38%) |
| B43 | <1 year | Male | UAE | 90 | 34.14 | 2.20 | ACTGAT | | M160108 | Alphacoronavirus (21, 10.66%), MERS-CoV (4207, 98.85%) |
| B44 | >1year | Male | UAE | 58 | 31.33 | 3.33 | GAATTCGT | TATAGCCT | M160304 | Alphacoronavirus (13, 5.77%), MERS-CoV (5, 2.97%), Bocaparvovirus (2, 1.52%) |
| B45 | <1 year | Male | OMAN | 58 | 20.63 | 2.31 | ATTCAGAA | GTACTGAC | M160304 | Alphacoronavirus (26397, 99.95%), MERS-CoV (11429, 99.84%) |
| B46 | >1year | Male | UAE | 54 | 29.93 | 9.11 | ATTCAGAA | CAGGACGT | 3 | Alphacoronavirus (211, 44.11%), MERS-CoV (467, 58.60%), Gemykrogrvirus (519, 54.21%) |
| B47 | <1 year | Male | UAE | 53 | 25.03 | 8.20 | ATTCAGAA | TAATCTTA | 3 | Alphacoronavirus (152873, 99.98%), MERS-CoV (8572, 99.91%), Gemykrogrvirus (34, 22.80%) |
| B48 | <1 year | Female | UAE | 58 | 23.58 | 3.28 | ATTCAGAA | AGGCGAAG | M160304 | Alphacoronavirus (8377, 99.93%), MERS-CoV (1498, 94.34%) |
| B49 | <1 year | Female | UAE | 58 | 30.79 | 4.09 | ATTCAGAA | GGCTCTGA | M160304 | Alphacoronavirus (177, 44.12%), MERS-CoV (50, 15.36%), Respirovirus (2253, 95.02%) |
| B50 | <1 year | Male | UAE | 55 | 24.8 | 9.34 | ATTCAGAA | CCTATCCT | 3 | Alphacoronavirus (96282, 99.98%), MERS-CoV (2594, 98.87%), camel coronavirus HKU23 (30, 7.62%) |
| B51 | <1 year | Male | UAE | 53 | 34.73 | 1.50 | CGTACG | | M160115 | Alphacoronavirus (1, 0.41%), MERS-CoV (691, 74.43%), Bocaparvovirus (4, 8.49%), Orthopoxvirus (2, 0.08%), Respirovirus (1, 0.63%) |
| B52 | <1 year | Male | UAE | 49 | 32.47 | 11.41 | ATTCAGAA | ATAGAGGC | 3 | Alphacoronavirus (52, 14.52%), MERS-CoV (262, 51.29%), Bocaparvovirus (2, 3.58%), Gemykrogrvirus (42, 27.78%) |
| B53 | <1 year | Male | UAE | 42 | 23.36 | 6.54 | ATTCAGAA | TATAGCCT | M160304 | MERS-CoV (3160, 99.83%), Respirovirus (3, 3.64%) |
| B54 | <1 year | Male | UAE | 76 | 32.46 | 5.27 | AGCGATAG | GTACTGAC | 2 | Alphacoronavirus (4, 1.80%), MERS-CoV (1, 0.36%), Bocaparvovirus (3, 6.63%) |
| B55 | <1 year | Female | UAE | 76 | 31.94 | 6.56 | AGCGATAG | CAGGACGT | 2 | Alphacoronavirus (23, 7.10%), MERS-CoV (12, 3.24%), Bocaparvovirus (1416, 73.91%), Orthopoxvirus (4, 0.15%), Parapoxvirus (1, 0.09%) |
| B56 | <1 year | Female | OMAN | 76 | 25.63 | 12.04 | AGCGATAG | TAATCTTA | 2 | MERS-CoV (298, 57.06%), Respirovirus (2, 1.36%) |
| B57 | <1 year | Male | OMAN | 76 | 33.1 | 6.13 | AGCGATAG | AGGCGAAG | 2 | MERS-CoV (10, 3.00%), Gemykrogrvirus (3, 11.87%) |
| B58 | <1 year | Male | OMAN | 76 | 33.45 | 9.98 | AGCGATAG | GGCTCTGA | 2 | Alphacoronavirus (20, 6.65%), MERS-CoV (2, 0.59%), Gemykrogrvirus (63, 86.02%), Respirovirus (4, 2.73%) |
| B59 | <1 year | Male | OMAN | 76 | 28.73 | 6.18 | AGCGATAG | CCTATCCT | 2 | Alphacoronavirus (1972, 94.79%), MERS-CoV (20, 6.08%), Gemykrogrvirus (1, 4.03%) |
| B60 | <1 year | Female | OMAN | 76 | 19.51 | 7.00 | AGCGATAG | ATAGAGGC | 2 | Alphacoronavirus (94, 28.10%), MERS-CoV (3189, 98.96%), camel coronavirus HKU23 (21, 4.44%), Bocaparvovirus (3, 5.06%), Respirovirus (1, 0.80%) |
| B61 | <1 year | Female | OMAN | 76 | 29.49 | 8.28 | AGCGATAG | TATAGCCT | 2 | Alphacoronavirus (14699, 99.91%), MERS-CoV (32, 9.70%), Gemykrogrvirus (4, 12.50%) |
| B62 | <1 year | Male | UAE | 76 | 18.6 | 5.60 | TCTCGCGC | GTACTGAC | 2 | Alphacoronavirus (29018, 99.98%), MERS-CoV (16448, 99.72%), Bocaparvovirus (4, 6.63%), Gemykrogrvirus (2, 9.59%) |
| B63 | <1 year | Male | OMAN | 76 | 19.12 | 4.72 | TCTCGCGC | CAGGACGT | 2 | Alphacoronavirus (59, 17.26%), MERS-CoV (4642, 99.59%), Gemykrogrvirus (3, 10.39%) |
| B64 | >1year | Female | OMAN | 76 | 19.28 | 4.15 | TCTCGCGC | TAATCTTA | 2 | Alphacoronavirus (2430, 97.02%), MERS-CoV (1343, 93.65%) |
| B65 | <1 year | Male | OMAN | 76 | 22.47 | 6.47 | TCTCGCGC | AGGCGAAG | 2 | Alphacoronavirus (3730, 99.13%), MERS-CoV (69434, 99.90%), Bocaparvovirus (47, 34.92%), Gemykrogrvirus (36, 45.25%), Respirovirus (47, 18.75%) |
| B66 | <1 year | Female | OMAN | 76 | 29.46 | 4.58 | TCTCGCGC | GGCTCTGA | 3 | Alphacoronavirus (50288, 99.97%), MERS-CoV (30, 9.38%) |
| B67 | <1 year | Male | OMAN | 76 | 17.06 | 3.82 | TCTCGCGC | CCTATCCT | 3 | Alphacoronavirus (18, 6.41%), MERS-CoV (8104, 99.93%), Gemykrogrvirus (3, 12.14%) |
| B68 | <1 year | Male | OMAN | 77 | 29.97 | 10.21 | TCTCGCGC | ATAGAGGC | 1 | Alphacoronavirus (66, 17.44%), MERS-CoV (152, 33.12%), camel coronavirus HKU23 (1, 0.37%), Bocaparvovirus (3, 4.13%), Gemykrogrvirus (23, 28.85%), Respirovirus (2, 0.79%) |
| B69 | <1 year | Male | OMAN | 77 | 19.82 | 8.21 | TCTCGCGC | TATAGCCT | 1 | Alphacoronavirus (100, 23.04%), MERS-CoV (287059, 99.92%), camel coronavirus HKU23 (1485, 85.34%), Respirovirus (9, 4.31%) |
| B70 | <1 year | Male | UAE | 77 | 23.54 | 11.38 | GAGATTCC | GTACTGAC | 1 | Alphacoronavirus (101, 23.04%), MERS-CoV (23329, 99.88%), Gemykrogrvirus (2, 10.22%), Kobuvirus (2, 1.69%) |
| B71 | <1 year | Female | OMAN | 77 | 25.97 | 6.45 | GAGATTCC | CAGGACGT | 1 | Alphacoronavirus (363, 58.15%), MERS-CoV (8870, 99.79%), camel coronavirus HKU23 (880, 74.73%), Bocaparvovirus (2, 4.24%), Gemykrogrvirus (48, 19.52%), Orthopoxvirus (3, 0.13%) |
| B72 | >1year | Male | UAE | 77 | 23.14 | 9.97 | TCCGCGAA | ATAGAGGC | 1 | Alphacoronavirus (76018, 99.97%), MERS-CoV (12222, 99.74%), Respirovirus (2, 1.43%) |
| B73 | <1 year | Male | OMAN | 77 | 17.76 | 8.92 | TCCGCGAA | TATAGCCT | 1 | Alphacoronavirus (998, 86.38%), MERS-CoV (90802, 99.90%), Gemykrogrvirus (5, 14.47%) |
| B74 | <1 year | Female | OMAN | 77 | 29.26 | 6.75 | CGGCTATG | GTACTGAC | 3 | Alphacoronavirus (69, 17.85%), MERS-CoV (187, 34.83%), Gemykrogrvirus (2, 4.95%) |
| B75 | <1 year | Male | OMAN | 77 | 28.99 | 9.55 | CGGCTATG | CAGGACGT | 3 | Alphacoronavirus (215, 43.59%), MERS-CoV (505, 66.96%), camel coronavirus HKU23 (108, 20.80%), Bocaparvovirus (5, 7.73%) |
| B76 | <1 year | Male | OMAN | 77 | 27.74 | 8.67 | CGGCTATG | TAATCTTA | 2 | Alphacoronavirus (33, 9.51%), MERS-CoV (95, 23.66%), Bocaparvovirus (7, 8.01%), Parapoxvirus (14, 1.00%) |
| B77 | <1 year | Male | UAE | 42 | 29.13 | 12.20 | CGGCTATG | AGGCGAAG | 2 | Alphacoronavirus (157, 36.16%), MERS-CoV (259, 46.03%), camel coronavirus HKU23 (2, 0.51%), Bocaparvovirus (13, 17.83%), Gemykrogrvirus (167, 23.90%), Orthonairovirus (22, 11.21%), Parapoxvirus (29, 1.64%) |
| B78 | <1 year | Male | UAE | 41 | 30.89 | 6.87 | CGGCTATG | GGCTCTGA | 2 | Alphacoronavirus (1468, 91.89%), MERS-CoV (12, 3.36%) |
| B79 | <1 year | Male | OMAN | 77 | 30.07 | 10.86 | CGGCTATG | CCTATCCT | 2 | Alphacoronavirus (91, 25.28%), MERS-CoV (202, 39.15%), Bocaparvovirus (4, 7.46%), Gemykrogrvirus (184, 54.66%), Parapoxvirus (4, 0.31%), Respirovirus (2, 0.90%) |
| B80 | <1 year | Female | OMAN | 77 | 29.71 | 12.42 | CGGCTATG | ATAGAGGC | 2 | Alphacoronavirus (148, 34.34%), MERS-CoV (63, 17.61%), Deltapapillomavirus (9, 10.57%), Gemykrogrvirus (4, 10.62%) |
| B81 | <1 year | Male | OMAN | 77 | 30.72 | 9.60 | CGGCTATG | TATAGCCT | 2 | Alphacoronavirus (486, 67.75%), MERS-CoV (71, 14.17%), Bocaparvovirus (2, 5.46%), Parapoxvirus (5, 0.37%) |
| B82 | <1 year | Male | OMAN | 77 | 30.75 | 12.42 | TAATGCGC | GTACTGAC | 2 | Alphacoronavirus (31, 9.57%), MERS-CoV (17, 4.72%), Bocaparvovirus (9, 10.70%), Gemykrogrvirus (11, 16.40%), Parapoxvirus (4, 0.23%) |
| B83 | <1 year | Female | UAE | 77 | 28.6 | 9.47 | TAATGCGC | CAGGACGT | 2 | Alphacoronavirus (675, 73.51%), MERS-CoV (1347, 88.73%), camel coronavirus HKU23 (5, 1.10%), Bocaparvovirus (9, 15.67%), Gemykrogrvirus (225, 21.50%), unclassified polyomavirus (53, 50.66%) |
| B84 | <1 year | Female | UAE | 43 | 29.97 | 12.48 | TAATGCGC | TAATCTTA | 2 | Alphacoronavirus (73, 20.66%), MERS-CoV (93, 22.22%), Deltapapillomavirus (2, 3.26%), Orthonairovirus (17, 9.19%), Parapoxvirus (3, 0.10%) |
| B85 | <1 year | Female | OMAN | 46 | 30.32 | 9.54 | TAATGCGC | AGGCGAAG | 2 | Alphacoronavirus (120, 29.64%), MERS-CoV (257, 42.60%), Bocaparvovirus (13, 16.98%), Gemykrogrvirus (137, 21.83%), Kobuvirus (4, 2.47%), Respirovirus (12, 6.05%) |
| B86 | <1 year | Male | OMAN | 47 | 31.38 | 9.70 | TAATGCGC | GGCTCTGA | 2 | Alphacoronavirus (42, 12.69%), MERS-CoV (5, 1.73%) |
| B87 | <1 year | Male | OMAN | 58 | 30.51 | 10.34 | TAATGCGC | CCTATCCT | 2 | Alphacoronavirus (135, 33.37%), MERS-CoV (56, 13.88%), Gemykrogrvirus (278, 92.70%), Parapoxvirus (1, 0.07%), Respirovirus (1, 0.69%) |
| B88 | <1 year | Male | OMAN | 58 | 29.94 | 11.80 | TAATGCGC | ATAGAGGC | 2 | Alphacoronavirus (39, 12.65%), MERS-CoV (10, 3.32%), Gemykrogrvirus (37, 61.83%) |
| B89 | <1 year | Male | UAE | 58 | 31.73 | 11.03 | TAATGCGC | TATAGCCT | 2 | Alphacoronavirus (32, 10.18%), MERS-CoV (10, 3.07%), Gemykrogrvirus (123, 100.00%), Rubulavirus (40, 12.94%) |
| B90 | <1 year | Female | UAE | 58 | 30.15 | 6.57 | CTGAAGCT | GTACTGAC | 2 | Alphacoronavirus (994, 86.76%), MERS-CoV (348, 54.42%), Bocaparvovirus (22148, 77.83%), Gemykrogrvirus (13, 23.16%) |
| B91 | <1 year | Male | UAE | 41 | 17.83 | 5.94 | CTGAAGCT | CAGGACGT | 2 | Alphacoronavirus (216, 45.73%), MERS-CoV (22676, 99.88%), Bocaparvovirus (5, 9.55%) |
| B93 | no info | no info | no info | no info | 25.4 | 9.99 | CTGAAGCT | AGGCGAAG | 2 | Alphacoronavirus (3921, 99.78%), MERS-CoV (1978, 93.45%), Bocaparvovirus (2, 4.30%), Gemykrogrvirus (3, 6.81%) |
| B94 | no info | no info | no info | no info | 31 | 7.39 | CTGAAGCT | GGCTCTGA | 2 | Alphacoronavirus (117, 29.56%), MERS-CoV (17, 4.52%), Bocaparvovirus (6, 13.96%), Orthopoxvirus (8, 0.46%) |
| B95 | >1year | Male | UAE | 42 | 28.87 | 8.47 | CTGAAGCT | CCTATCCT | 2 | Alphacoronavirus (109, 31.07%), MERS-CoV (515, 60.96%), Bocaparvovirus (8, 15.38%), Deltapapillomavirus (1, 3.26%), Parapoxvirus (5, 0.21%), Respirovirus (2, 0.65%) |
| B96 | no info | no info | no info | no info | 25 | 6.74 | CTGAAGCT | ATAGAGGC | 2 | Alphacoronavirus (474140, 99.96%), MERS-CoV (35825, 99.80%), Bocaparvovirus (9, 8.00%) |
| B97 | >1year | Female | UAE | 43 | 22.84 | 6.95 | CTGAAGCT | TATAGCCT | 2 | Alphacoronavirus (301, 54.61%), MERS-CoV (89288, 99.94%), Gemykrogrvirus (26, 42.07%) |
| B99 | >1year | Male | OMAN | 77 | 30.23 | 14.59 | GAATTCGT | GTACTGAC | 3 | Alphacoronavirus (41, 12.38%), MERS-CoV (252, 35.48%), Bocaparvovirus (42, 26.32%), Deltapapillomavirus (1, 2.88%), Gemykrogrvirus (14, 16.78%), Orthonairovirus (17, 8.86%), Parapoxvirus (7, 0.50%) |
| B100 | <1 year | Male | OMAN | 77 | 30.27 | 6.41 | GAATTCGT | CAGGACGT | 3 | Alphacoronavirus (685, 76.36%), MERS-CoV (385, 57.32%) |
| B101 | >1year | Female | OMAN | 77 | 27.56 | 14.59 | GAATTCGT | TAATCTTA | 3 | Alphacoronavirus (11928, 99.92%), MERS-CoV (701, 70.08%), camel coronavirus HKU23 (85469, 99.89%), Bocaparvovirus (8, 15.38%) |
| B102 | >1year | Male | OMAN | 77 | 28.62 | 10.37 | GAATTCGT | AGGCGAAG | 3 | Alphacoronavirus (30, 8.51%), MERS-CoV (1716, 91.21%) |
| B103 | <1 year | Male | OMAN | 77 | 22.21 | 7.67 | GAATTCGT | GGCTCTGA | 3 | MERS-CoV (19486, 99.82%), Parapoxvirus (4, 0.22%) |
| B104 | <1 year | Female | OMAN | 77 | 28.75 | 7.52 | GAATTCGT | CCTATCCT | 3 | Alphacoronavirus (40, 12.40%), MERS-CoV (128, 29.22%), Bocaparvovirus (2, 8.28%) |
| B105 | <1 year | Female | OMAN | 77 | 26.86 | 6.96 | GAATTCGT | ATAGAGGC | 3 | Alphacoronavirus (26, 7.13%), MERS-CoV (1210, 88.28%), camel coronavirus HKU23 (24, 5.28%), Bocaparvovirus (37, 34.18%) |
| B106 | <1 year | Male | OMAN | 77 | 21.22 | 8.04 | GAATTCGT | TATAGCCT | 3 | MERS-CoV (8583, 99.79%), Bocaparvovirus (1, 1.61%) |
| B107 | <1 year | Male | OMAN | 77 | 31.02 | 5.36 | ATTCAGAA | GTACTGAC | 3 | Alphacoronavirus (28046, 99.99%), MERS-CoV (16, 4.54%), Gemykrogrvirus (3, 10.48%), Parapoxvirus (2, 0.15%) |
| B108 | <1 year | Female | OMAN | 77 | 30.78 | 7.51 | ATTCAGAA | AGGCGAAG | 3 | Alphacoronavirus (537, 64.17%), MERS-CoV (382, 52.73%), Gemykrogrvirus (229, 86.74%) |
| B109 | <1 year | Male | UAE | 77 | 30.43 | 8.07 | ATTCAGAA | GGCTCTGA | 3 | Alphacoronavirus (87, 22.06%), MERS-CoV (41, 11.14%), Gemykrogrvirus (30, 30.06%) |
| B110 | >1year | Male | OMAN | 77 | 30.87 | 5.64 | ATTCAGAA | TATAGCCT | 3 | Alphacoronavirus (1224, 88.94%), MERS-CoV (11, 2.86%), Bocaparvovirus (2, 2.14%), Gemykrogrvirus (11, 42.29%) |
| B111 | <1 year | Male | UAE | 77 | 30.7 | 11.04 | TCCGCGAA | GTACTGAC | 3 | Alphacoronavirus (64, 17.00%), MERS-CoV (42, 11.04%), camel coronavirus HKU23 (8, 2.17%), Gemykrogrvirus (14, 28.27%) |

* Genus names are used for most viruses as there was one virus per genus, except for beta-CoV genus where two viruses were identified and are addressed separately.
