# Supplementary material for: Identification of diverse viruses in upper respiratory samples in dromedary camels from United Arab Emirates
Source: PLoS One. 2017 Sep 13;12(9):e0184718. doi: 10.1371/journal.pone.0184718 (PMC5597213; doi:10.1371/journal.pone.0184718)
Supplement: S2 Table — (PDF) [file pone.0184718.s003.pdf]

S2 Table. PCR primers used for viruses detected in this study

Virus	Virus group	Primer name and sequence (5'-3')	Reference
Camel alpha-CoV Abu Dhabi	Coronaviruses	**Forward**: F2: ATGGGITGGGAYTATCCWAARTGTG; **Reverse**: R2: CTAGTICCACCIGGYTTWANRTA	[34]
Camel CoV HKU23 Abu Dhabi	Coronaviruses	**Forward**: F2: ATGGGITGGGAYTATCCWAARTGTG; **Reverse**: R2: CTAGTICCACCIGGYTTWANRTA	[34]
Camel bocavirus 3 Abu Dhabi	Bocaparvoviruses	**Forward**: Boca-F1730: TGTTCGGCTITATGGITGYGTNAAYCA; **Reverse**: Boca-R2055: CCAAATGTGTGTGGIAGTTGYTTCATRAARTT	n/a
Camel CCHFV Abu Dhabi	Orthonairoviruses	**Forward**: F7145: GCTTCTATGCAGTTTTAGCACCIAARGCNCA; **Reverse**: R7447: TGTATCGGGCCCCATTTIGTRTTRTCNCC	n/a
Camel PIV3 Abu Dhabi	Paramyxoviruses	**Forward**: RES-MOR-HEN-F1: TCITTCTTTAGAACITTYGGNCAYCC; **Reverse**: RES-MOR-HEN-R: CTCATTTTGTAIGTCATYTTNGCRAA	[35]
Camel PIV4 Abu Dhabi	Paramyxoviruses	**Forward**: AVU-RUB-F2: ACACTCTATGTIGGIGAICCNTTYAAYCC; **Reverse**: AVU-RUB-R: GCAATTGCTTGATTITCICCYTGNAC	[35]
Camel polyomavirus Abu Dhabi	Polyomaviruses	**1st Rd: Forward**: PYV-05-01 F0: GGAAATCTACTGATGTGGGARGCNRT; **Reverse**: PYV-05-06 R2: CAAAGIGGCCCAACNCCATNYTCAT	[36]
		**2nd Rd: Forward**: PYV-05-01 F0: GGAAATCTACTGATGTGGGARGCNRT; **Reverse**: PYV-05-05 R1-1: GTTCCAAAATATCTNGTRTTYTCATT	[36]
Camel AAV	Dependoparvoviruses	**Forward**: dependo-F745: CCTCGGGI RATTGGCAYTGYGA; **Reverse**: dependo-R1000: GTTGTTGATGAGNCKYTGCCARTC	n/a
Dromedary astrovirus	Astroviruses	**Forward**: Ast-F1: TGGACCCGCTATGATGGIACNATHCC; **Reverse**: Ast-R1: TTCCCAGGCTTIACCCACATRCCRAA	n/a
